# Current pattern of antibiotic resistance of clinical isolates among conjunctival swabs

**DOI:** 10.12669/pjms.291.2566

**Published:** 2013

**Authors:** Farhan Essa Abdullah, Mariya Irfan Khan, Sadia Waheed

**Affiliations:** 1 Farhan Essa Abdullah, MBBS, MPhil, PhD (Microbiology), Assistant Professor of Microbiology and Immunology, Dr. Essa’s Laboratory & Diagnostic Center, Karachi.; 2 Mariya Irfan Khan, Final Year MBBS, Sindh Medical College, Karachi, Pakistan.; 3 Sadia Waheed, Final Year MBBS, Sindh Medical College, Karachi, Pakistan.

**Keywords:** Conjunctival swab, gram positive bacteria, empirical therapy

## Abstract

***Objectives:*** To identify the etiological agent in bacterial conjunctivitis and to determine the antibiogram of bacterial isolates.

***Methodology:*** This observational study was conducted at Dr. Essa’s Laboratory over a period of 12 months ending in March 2012. Two hundred samples taken from conjunctiva of patients with conjunctivitis were cultured on routine medium and the antibiograms of bacterial isolates were determined by Kirby- Bauer disc diffusion method.

***Results: ***The analysis of the culture showed that 41% were cultured positive with gram positive bacteria *Staphylococcus aureus* 52.5% and *Staphylococcus epidermidis *30.1% and *Micrococci *8.3%. However, 9.1% were gram negatives with *Klebsiella pneumoniae *5.14% and *Pseudomonas aeruginosa* 2.6% *and 1.36% *were others* (Acinetobacter, Haemophilus* , *E.coli and Moraxella*) keeping in view the increasing use of contact lens and unclean fingers. The overall antibiograms of bacterial isolates indicate aminoglycosides (gentamicin, tobramicin) and the newer quinolones as apparent drug of choice for empirical therapy, followed by chloramphenicol, since drug fussy gram-negatives such as *Pseudomonas, Acinetobacter* and *E.coli* were among the conjunctival isolates. Resistance profile of gram positive isolates shows cefixime 91.4%, doxycycline 57.9%, cotrimoxazole 29.3%, ampicillin 22.9%, ciprofloxacin 13.4%, cephradine 8.3%, cefuroxime 7.1%, fosfomycin 4.7%, ceftriaxone 3.6%, co-amoxiclav 3.6%, cefotaxime 3.5%, vancomycin 2.6%.

***Conclusion: ***Resistance to all conventionally used antibiotics is increasing, therefore identification of etiological agent and antibiogram is important to treat conjunctivitis and to avoid complications.

## Introduction

 Conjunctivitis is so common all over the world that almost everyone has experienced it at least once in a lifetime. It is one of the most frequently reported disease in the outpatient and emergency departments.^1^

 Conjunctiva is a mucous membrane that covers the sclera (bulbar conjunctiva) and lines the inside of the eyelid (palpebral conjunctiva). It consists of a non-keratinized stratified squamous epithelium with blood vessels and lymphoid tissue. It has a major protective function in guarding against infectious organisms and provides surveillance for antigenic stimuli.^2^ Therefore, infection and inflammation of conjunctiva may impair its protective function resulting in contiguous infections. 

 Aetiology of conjunctivitis can be categorized into two broad categories namely infectious (bacteria, virus or fungi) and non-infectious (allergic, mechanical, chemical etc).^3^ Infectious conjunctivitis is mainly bacterial or viral, with approximately 78% to 80% of cases being bacterial in origin.^4^ Common routes of transmission involves contact exposure to airborne fomites, skin flora on hands, upper respiratory infections, or genital secretions. The general symptoms include congestion and swelling of eye with a gritty feel and irritation due to sticky discharge. Eyelids may fuse together especially in morning making it difficult to open.^3^ If untreated, conjunctivitis can cause severe morbid sequelae like blindness, septicemia, meningitis, cellulitis and even otitis media. *Staphylococcus aureus *causes recurrent conjunctivitis associated with chronic blepharoconjuntivits.^3^

 Self limitation is generally seen in bacterial conjunctivitis. However a meta-analysis has revealed that use of topical antibiotics shorten the course of disease and duration of symptoms^5^^,^^6^, reduces transmission risk and abates the chances of ocular and extra ocular complications.^4^

 Patient’s history and examination are sometimes insufficient for diagnosis and treatment, thus culture of conjunctival swabs is integral in identification of causative pathogens.^2^ While prescribing an antibiotic, the physician should have in mind the most likely pathogen and the cost and side effects of each drug.^4^ Antibiotics are considered to be the most important lifeline in the field of medicine but unfortunately with each passing decade, bacteria are developing resistance with not only a single but multiple antibiotics. The rise of bacterial resistance is highly promoted by misuse of antibiotics and has thus become a serious challenge to the field of medicine in its fight against bacterial infections.^7^

 Therefore our study aimed to identify the common etiological agents in conjunctivitis and their antibiograms for effective treatment and avoidance of complications.

## Methodology

 An Observational study was conducted at Dr. Essa’s Laboratory and Diagnostic Centre, Karachi, for a period of 12 months ending in March 2012. A total of 200 samples were taken from conjunctiva of patients with conjunctivitis. Consent was taken from the patients for using the results of their swab cultures in our research. Conjunctival swabs were collected under sterile conditions in order to prevent contamination of samples. They then underwent Gram-stain microscopy followed by inoculation onto CLED, EMB, Blood and Chocolate agars. Gram positive and gram negative isolates were then identified using API kit. Finally antibiograms of bacterial isolates were determined by Kirby- Bauer disc diffusion method.

**Table-I:** Bacterial isolates in Conjunctival swabs

*Bacterial Isolates*	*%*
Gram Positive Bacteria	
Staphylococcus aureus	*52.5%*
Staphylococcus epidermidis	*30.1%*
Micrococci	*8.3%*
Gram Negative Bacteria	
Klebsiella pneumoniae	*5.14%*
Pseudomonas aeruginosa	*2.6%*
Others (Acinetobacter, Haemophilus, E.coli , Moraxella)	*1.36%*

## Results

**Fig.1:**
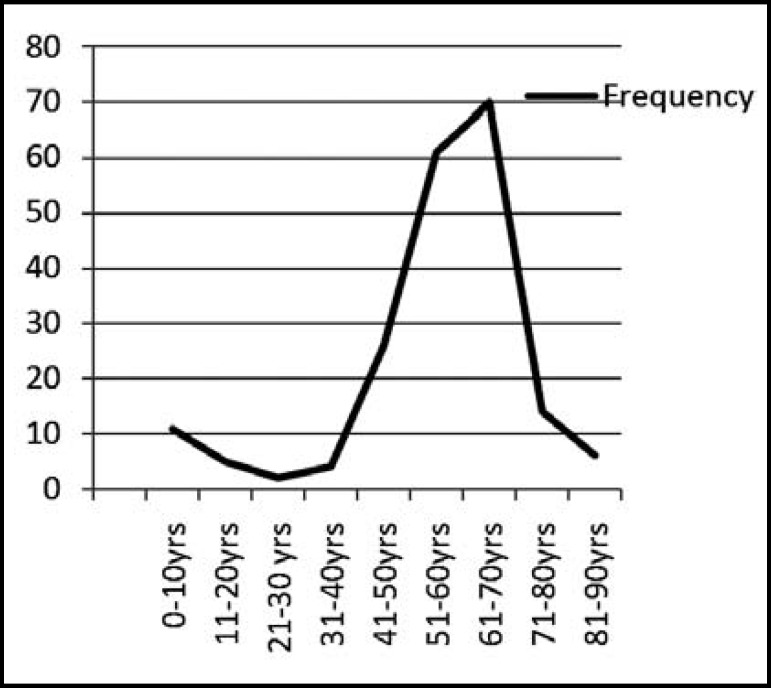
Figure Showing Frequency of Age groups.

 Out of 200 conjunctival swabs of patients with conjunctivitis 41% were cultured positive. The isolates identified in culture are shown in Table-I. Our analysis showed a high frequency of conjunctivitis among females 61% (n=122) and rest were males 39% (n=78). The majority of cases were noted between 41 to 70 years (79.22%) with peak incidence in age group between 61 to 70 years (34.4%) as shown in Fig.1.

**Table-II:** Antibiotic Sensitivity Profile for Gram Positive and Gram Negative Isolates

*Antibiotics*	*Sensitivity profile for Gram Positive isolate*	*Sensitivity profile for Gram Negative isolate*
Gentamicin	86.7%	80%
Tobramycin	84.5%	100%
Sparfloxacin	86.8%	100%
Moxifloxacin	86.3%	100%
Chloramphenicol	81.7%	75%

 The overall antibiograms of bacterial isolates indicate quinolones (sparfloxacin and moxifloxacin) and aminoglycosides (gentamicin, tobramycin) as apparent drug of choice for empirical therapy, followed by chloramphenicol, since drug fussy gram-negatives such as *Pseudomonas, Acinetobacter* and *E.coli* were among the conjunctival isolates. This is shown in Table-II. Antibiotic resistance and sensitivity profile of Gram-positive isolates is shown in Table-III.

## Discussion

 In our study, *Staphylococcus aureus *was found to be the major cause of bacterial conjunctivitis which is in accordance to the existing literature.^2^^,^^3^^,^^8^ Our result also demonstrated *Klebsiella pneumonia* to be the major gram negative isolate in conjunctivitis but previous studies have indicated *Haemophilus influenza* to be the major culprit.^2^^,^^3^ According to another research in India the prevalence of bacterial conjunctivitis was found to be 20.4% with a predominance of *Staphylococcus aureus* (87.2%) followed by *Streptococcus* *pneumoniae* (4.7%) and gram negative rods (*E.coli* , *Klebsiella.*, *Pseudomonas* ) in 8.1% swabs.^8^

 Coagulase-negative *Staphylococci (CoNS)* reported in our result was previously ignored to cause severe infections. As a result of the combination of increased use of intravascular devices and an increase in the number of hospitalized immunocompromised patients, *CoNS *have emerged as a major cause of nosocomial bloodstream infections.^9^

 Our study indicates that conjunctivitis is more common among elderly with 34.4% belonging to age group between 61 to 70 years. They are at high risk of infections due to poor cell mediated immunity, malnutrition^10^ and also comorbidities prevailing in their lives.

 It was interesting to note that females (61%) were more prone to conjunctivitis which was on the contrary to the study of Puja et al in which males outnumber females by 64%.^11^ According to another study in South Florida, male and female patients contributed equal percentages to the total isolates.^12^ This gender variation may vary on region to region basis.

 According to Everett et al, the percent susceptibility of recovered isolates to single antibiotic agents or combinations were ranked from greatest to least: chloramphenicol, bacitracin/polymyxin B, ofloxacin, sulfonamide, ciprofloxacin, trimethoprim/polymyxin B, norfloxacin, gentamicin, bacitracin, trimethoprim, tobramycin, neomycin, erythromycin, and polymyxin B and it was noted that none of the available topical antibiotics provided 100% broad spectrum coverage in vitro.^13^ Egger et al also demonstrated that chloramphenicol had the highest overall in vitro efficacy and in their study the relative overall in vitro efficacy was (in decreasing order): chloramphenicol, ciprofloxacin, ofloxacin, norfloxacin, bacitracin, tetracycline, neomycin, erythromycin, tobramycin and gentamicin.^14^ On the contrary our result showed that chloramphenicol did not show a satisfactory efficacy with only 81.7% sensitive to gram positive and 75% sensitive to gram negative isolates.

**Table-III:** Antibiotic Resistance and Sensitivity Profile of Gram-positive isolates

*Antibiotics*	*Resistance (%)*	*Sensitivity (%)*
Cefixime	91.4	6.2
Doxycycline	57.9	31.6
Cotrimoxazole	29.3	61.0
Ampicillin	22.9	67.5
Ciprofloxacin	13.4	76.8
Cephradine	8.3	85.7
Cefuroxime	7.1	90.5
Fosfomycin	4.7	89.4
Ceftriaxone	3.6	90.4
Co-amoxiclav	3.6	95.2
Cefotaxime	3.5	90.6
Vancomycin	2.6	97.4

 Quinolones (moxifloxacin and sparfloxacin) seems to be a better choice for empirical therapy.^15^^,^^16^ Moxifloxacin is a fourth generation fluoroquinolone that inhibits both DNA gyrase and topoisomerase IV. This provides enhanced activity against gram positive and gram negative organisms. Bacterial resistance against fluoroquinolones has been reported in systemic treatment but not in topical use. Topical use results in antibacterial concentrations at the ocular surfaces that exceed mutant prevention concentrations, and in the case of moxifloxacin a dual step mutation is required for resistance. Therefore, moxifloxacin is superior to other antibiotics in the management of bacterial conjunctivitis by reducing the disease spread and providing faster recovery.^17^ Out of all the empirical drugs listed in our result, moxifloxacin seems to be a better broad spectrum antibiotic with 86.3% sensitivity for gram positive isolates and 100% sensitivity to gram negative isolates but still its resistance to gram positive isolates cannot be ignored. Sparfloxacin shows a similar result.

 Vancomycin has always been recognized as one of the most potent drugs available against gram positive isolates.^17^ According to S. Hafiz et al no vancomycin resistant* Staphylococcus* has been isolated from any of the major cities.^18^ Also Adebukola et al did not find any resistance of gram positive pathogens to vancomycin^2^ but our study shows the resistance of vancomycin against gram positive isolates as 2.6%. Even minor resistance of gram positive isolates to vancomycin is a major concern because it is considered as the last resort for the resistant gram positive isolates.

## Conclusions

 A clear cut female predominance in our result raises a concern and therefore proper care should be sought as soon as possible with essential hygienic precautions including hand hygiene for the control of cross-transmission of resistant bacteria. Beneficial empirical therapies, according to our findings are quinolones (moxifloxacin and sparfloxacin) and aminoglycosides (gentamicin, tobramicin) as apparent drug of choice. Though vancomycin shows the highest efficacy against gram positive isolates even its slight emergence of resistance should be taken as a serious threat.
